# Case of the Successful Use of Mercury in Typhus; with Observations on Its Use in Tetanus.—On the Use of Cold Bath, in Rousing the Excitability of the System after Fever

**Published:** 1808-02

**Authors:** James Mease


### *Case of the successful Use of Mercury in Typhus; with Observations on its Use in Tetanus.—On the Use of Cold Bath, in rousing the Excitability of the System after Fever.* By JAMES MEASE, M. D.

THE publication of single cases of disease unconnected with the developement of principles in medicine, does not often serve a useful purpose; because, when such principles are well laid by the medical man, the application of them to practice is easy; and he who attempts to relieve the maladies of his fellow creatures, without a just and competent knowledge of them, and depends upon finding in books, cases recorded exactly similar to those he may be called to, may hunt without success, nineteen times out of twenty. A dependance upon particular forms of prescriptions is equally absurd, because, supposing the case required, to be found, it is more than probable that the difference in the constitution of the patient, may render the attempt at a cure by the same proportions of the medicines in both cases completely fruitless; hence the absolute, the indispensable necessity of a practical knowledge of pharmacy to the practitioner, by which I mean an attendance and personal labour, either in the shop of the apothecary, or in one of a physician who compounds his his own prescriptions; and hence the evident advantage which that physician will always have, who has risen gradually, (if rising it may be called) from the pestle and mortar, to the honour of feeling pulses, over the mere room student, or hospital-walker, who looks on, fearful of soiling his hands, and who, when he prescribes a box of pills, or compound mixture, or even superintends their composition, may be unable to say positively, whether they are rightly made up or not.

In some cases, however, a statement of the utility of a remedy in a particular case, may be highly advantageous to the practitioner, who will of course carefully treasure up the fact, and apply it with success in a similar case, under his own care, provided no objection occur to its use; and in this view, I communicate the two following cases, which fell under my care some time since.

*Cure of Typhus by Mercury.*

A sailor was landed at the Lazaretto, from the West-Indies, where he had laboured under a fever which terminated in an obstinate typhus, and had long resisted the usual remedies. I put him on the use of bark and wine without delay, with generous nourishment; but no effect was produced, nor was any change observed in his disease until after three weeks, when he became evidently more exhausted. I then gave him four grains of calomel combined with opium three times a-day; but the doses of calomel proving too large for his bowels, I determined to impregnate his system with mercury by external inunction. I accordingly had one ounce of strong ointment well rubbed into his lower extremities, three times a-day; for the rapidity with which he appeared to decline, the length of time his disease had subsisted, and the insensibility of his system, would not justify the slow and feeble use of this remedy; and I well knew, that unless the action of his system could be changed, he must continue to sink. In three days his mouth became slightly touched, and every unfavourable symptom disappeared. His constant drink was wine and water.

A circumstance rather singular, observed, was, that after the mercury had been discontinued some days, a salivation came on, and continued pretty severe for three or four days, and then ceased.

Several similar cases of long protracted typhus from the West Indies, occurred during my residence at the Lazaretto, which I treated with like success by mercury. The quantity required to affect the system when externally applied, plied, was sometimes immense; in one case, nearly three pounds of ointment were consumed, and I almost despaired of producing any effect by it, when a salivation at last came on, and cured the patient.

Though the propriety of the use of bark and wine in cases of great debility are certainly obvious, yet it is to be regretted that the insensibility of the system is often so great, as either not to be overcome by their exhibition, or to require a tedious use of them, which in some cases is attended with an expense that is very inconvenient. Instances of this insensibility of the system to internal stimuli, are in no cases more evident than those in the tetanus, in which disease, almost incredible quantities of wine have been taken before a cure was effected. The late Dr. Currie, of Liverpool, has related the case of a man, who, when ill with the disease in question, took 110 bottles of wine, and observes, that "ebriety was not produced; it soothed the irritation of the nerves, and comforted the mind, and without increasing the frequency of the pulse, it augmented it in strength."

A delicate young lady of Philadelphia, in typhus, began with one table-spoonful of Madeira wine, and proceeded to two in a day, and before she was cured, consumed 127 bottles. Where mercury may be given, it is certainly better to try it, whether we consider economy, the rapidity effected in the cure, or the injury which the constitution is saved from, by cutting short an enervating and alarming disease. Typhus in particular, is notorious for its injurious effects on both mind and body, produced by its long continuance, and it is in this disease, that the use of mercury promises the greatest benefit; for tetanus in *general* so soon terminates its course, that time is not given for the mercury to take effect, unless administered on the attack; and as the practitioner is seldom called until the disease is fully formed, a chance for its useful exhibition is not given. It might be well, however, to give it this chance, by immediately applying it externally and internally as soon as called, while at the same time other means are used.

In applying mercury for tetanus, the ointment should be rubbed in liberally to the whole body, which may be stripped and put between blankets, but it ought to be especially well rubbed in the throat, with a view to expedite the effect of salivation, and that the oil of the ointment may relax the rigid muscles.

*On the Utility of the Cold Bath.*

The mate of a vessel had been attacked by a fever in the the island of St. Domingo, about two months before I saw him. He recovered so far, as to be able to sail for Savannah, but was confined to his bed during the whole voyage. He remained at that place about two weeks, and arrived at the Lazaretto in the latter end of April. He was unable to walk, and could not speak above his breath; his face was swelled, countenance sallow, and he laboured under a violent headach. I now learned that he had been very intemperate before, and during his indisposition: his pulse was weak and soft; bowels regular. I applied a blister to the back of his neck, and gave him some cordial invigorating remedies, prescribing at the same time a meat diet, soup, with half a pint of wine per day. The blister relieved his head, and after a few days he appeared to mend slowly; but he had an insensibility of system that was very discouraging. His excitability appeared to be exhausted by his intemperance, and the long continuance of febrile indisposition under which he had laboured. I raised my stimuli in hopes to excite his system to healthy action, but in vain.

In about two weeks after I first saw him, he evidently began to decline; his pulse became weak; nervous tremors of his hands came on, and he required to be roused when spoken to, in order to obtain an answer. I applied blisters to his wrists and to the back of his neck, and directed three buckets of cold water to be dashed upon his naked body after being placed in a large bathing tub, and his body to be well rubbed by two persons with coarse towels, after the affusion of the water, and put between blankets. The good effects of this application were perceived in the course of half an hour, by the account he gave of the alteration in his feelings, which he said were unusually pleasant. He slept well that night, and in the morning early, I found him much better; indeed upon my entering the room, I pronounced him better from the evident change that appeared in his countenance; his eye was lively and clear, and he spoke with a strength and rapidity which truly surprised and pleased me. I immediately ordered the cold bath to be repeated, at which he expressed his satisfaction, and so great was the addition to his vigour from the first application, that he threw off the bed-clothes, and got into the tub. The bath was repeated for three days in succession with increasing benefit; but was discontinued by reason of a swelling which then appeared in his cheek. By the use of the tincture of gum-guaicum and generous diet he soon recovered.

